# Effect of warming regimen on canine epididymal spermatozoa following vitrification using a coconut water extender supplemented with egg yolk

**DOI:** 10.1186/s13028-026-00886-1

**Published:** 2026-10-09

**Authors:** Boyana Ivanova, Radka Garnoeva, Rumen Roydev, Anton Antonov

**Affiliations:** 1 Department of Obstetrics, Reproduction and Reproductive Disorders, Faculty of Veterinary Medicine, Trakia University, Stara Zagora, (6000) Bulgaria; 2 Department of Veterinary Surgery, Faculty of Veterinary Medicine, Trakia University, Stara Zagora, (6000) Bulgaria

**Keywords:** Coconut water, Dog, Egg yolk, Epididymis, Spermatozoa, Vitrification

## Abstract

**Background:**

Preservation of canine genetic material through epididymal sperm recovery and vitrification has gained increasing importance in breeding programs and germplasm conservation. The warming procedure represents a critical step affecting post-vitrification sperm survival and functionality. This study evaluated the effect of two warming temperatures on the quality of vitrified canine epididymal spermatozoa.

**Methods:**

Epididymal spermatozoa were collected from twelve clinically healthy dogs undergoing routine orchiectomy. Samples were vitrified using a coconut water-based extender supplemented with egg yolk and subsequently warmed at either 37 °C or 42 °C for 2 min. Sperm quality was evaluated before vitrification and after warming by assessment of motility, viability, morphology, and kinematic parameters using computer-assisted sperm analysis (CASA).

**Results:**

Vitrification significantly reduced sperm quality compared with fresh samples. However, warming at 37 °C resulted in significantly higher total motility, viability, and percentage of morphologically normal spermatozoa compared with warming at 42 °C. Several kinematic parameters were also better preserved following warming at 37 °C.

**Conclusions:**

The warming protocol significantly influences post-vitrification quality of canine epididymal spermatozoa. Warming at 37 °C for 2 min provided superior preservation of sperm function compared with warming at 42 °C and may represent a practical approach for canine germplasm banking and assisted reproduction.

## Introduction

Semen cryopreservation represents one of the most widely used assisted reproductive biotechnologies in male animals [1]. Two principal approaches to sperm freezing have been developed: conventional slow freezing and vitrification. Conventional cryopreservation frequently yields variable post-thaw survival due to suboptimal sperm quality after thawing, whereas vitrification involves ultra-rapid solidification of a liquid into a glass-like state by direct immersion into liquid nitrogen (LN₂), thereby preventing intracellular ice crystal formation [2–5]. Initially established for the preservation of embryos, oocytes, and tissues [2, 6, 7], vitrification has subsequently been successfully adapted for sperm preservation in multiple species including humans [8], fish [9], salmon [10], mouflon [11, 12], felids [13], donkey [14], and stallion [15]. Although conventional cryopreservation methods remain predominant in dogs, vitrification has emerged during the last decade as a promising alternative technique [16–24].

The dog is among the most common companion animals worldwide [25]. In recent years, semen cryopreservation has gained increasing importance in canine breeding, allowing both short- and long-term storage of spermatozoa, particularly for international genetic exchange [26]. Most preservation protocols have been developed for ejaculated semen, whereas comparatively few studies have investigated epididymal spermatozoa. Cryopreservation of epididymal sperm may be particularly valuable for conserving genetic material from animals that die unexpectedly, undergo orchiectomy for medical reasons, or belong to endangered wild canid species [20, 27].

Selection of an appropriate semen extender is a critical step in sperm preservation, as extender components must protect spermatozoa against cryoinjury [28, 29]. Numerous commercial extenders and cryoprotectants have been described for canine semen, and considerable attention has been directed toward natural biological additives. Coconut water represents one of the most extensively studied natural extender components [30–34]. It is isotonic, non-toxic, inexpensive, and easy to obtain [30], possesses strong antioxidant properties [30, 32, 35, 36], and contains phytohormones [37], sugars, vitamins, electrolytes, and amino acids [38]. Most studies using coconut water have focused on conventional cryopreservation of ejaculated semen, whereas only a few investigations, including those conducted by our research group, have evaluated its use in vitrification [21–24]. However, its potential application for vitrification of canine epididymal spermatozoa has not yet been reported.

To reduce cryoinjury, inclusion of a cryoprotectant is essential. Egg yolk is one of the most commonly used cryoprotectants, particularly in conventional freezing protocols. It protects spermatozoa from cold shock [39, 40], inhibits premature capacitation [41], and increases extender viscosity, thereby reducing water crystallization and ice formation [8]. Nevertheless, limited information exists regarding its effectiveness during ultra-rapid cryopreservation, especially in epididymal canine spermatozoa.

Among the factors affecting survival after vitrification, the warming procedure (devitrification) is critically important [11, 18, 42]. Previously described warming protocols for vitrified canine spermatozoa can be broadly categorized as slow warming (37 °C or 42 °C for 1–2 min) and rapid warming (approximately 65 °C for 2–5 s) [16, 18, 21–24]. It has been suggested that warming rate may be as crucial as cooling rate in vitrification [42], and slow warming may help prevent cellular damage in vitrified canine spermatozoa [18]. However, the influence of different slow-warming temperatures on vitrified canine epididymal spermatozoa has not yet been investigated.

Therefore, the aim of the present study was to evaluate the suitability of a coconut water extender supplemented with egg yolk for preservation of physiological parameters of canine epididymal spermatozoa during vitrification and to determine whether optimization of the slow-warming regimen improves post-thaw recovery.

## Materials and methods

### Ethics statement

Epididymal samples were obtained from dogs undergoing routine orchiectomy for clinical purposes unrelated to this study. Instead of being discarded, tissues were used for research. Therefore, approval from the Local Animal Ethics Committee was not required. Dog owners were informed about the study and provided written consent for use of the samples and publication of the results prior to castration.

#### Animals and sample collection

Twenty-four testes were collected from twelve clinically healthy dogs undergoing elective castration at the University Veterinary Hospital, Faculty of Veterinary Medicine, Trakia University, Stara Zagora, Bulgaria. Dogs belonged to different breeds (German Shepherd, *n* = 3; Beagle, *n* = 3; English Cocker Spaniel, *n* = 2; mixed breed, *n* = 4) and were aged between 1 and 4 years.

**Fig. 1:**
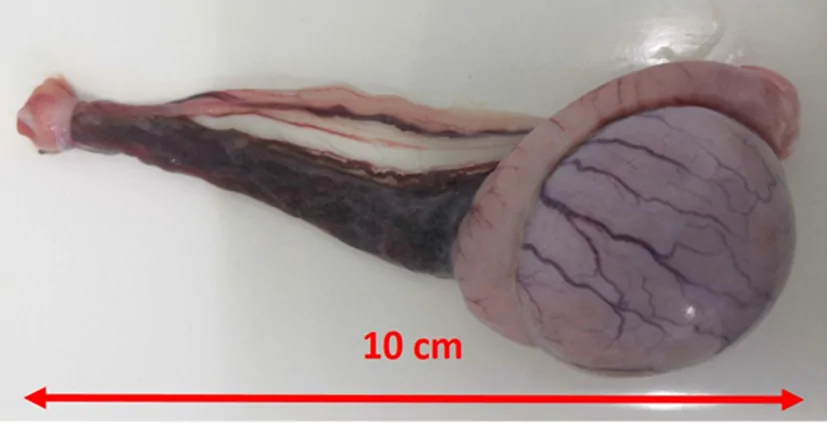
Canine testis with the epididymis after orchiectomy

Immediately after orchiectomy, testes and epididymides were placed in 50 mL plastic tubes containing saline pre-warmed to 37 °C and transported to the laboratory within 1 h (Fig. 1).

#### Epididymal sperm recovery

**Fig. 2:**
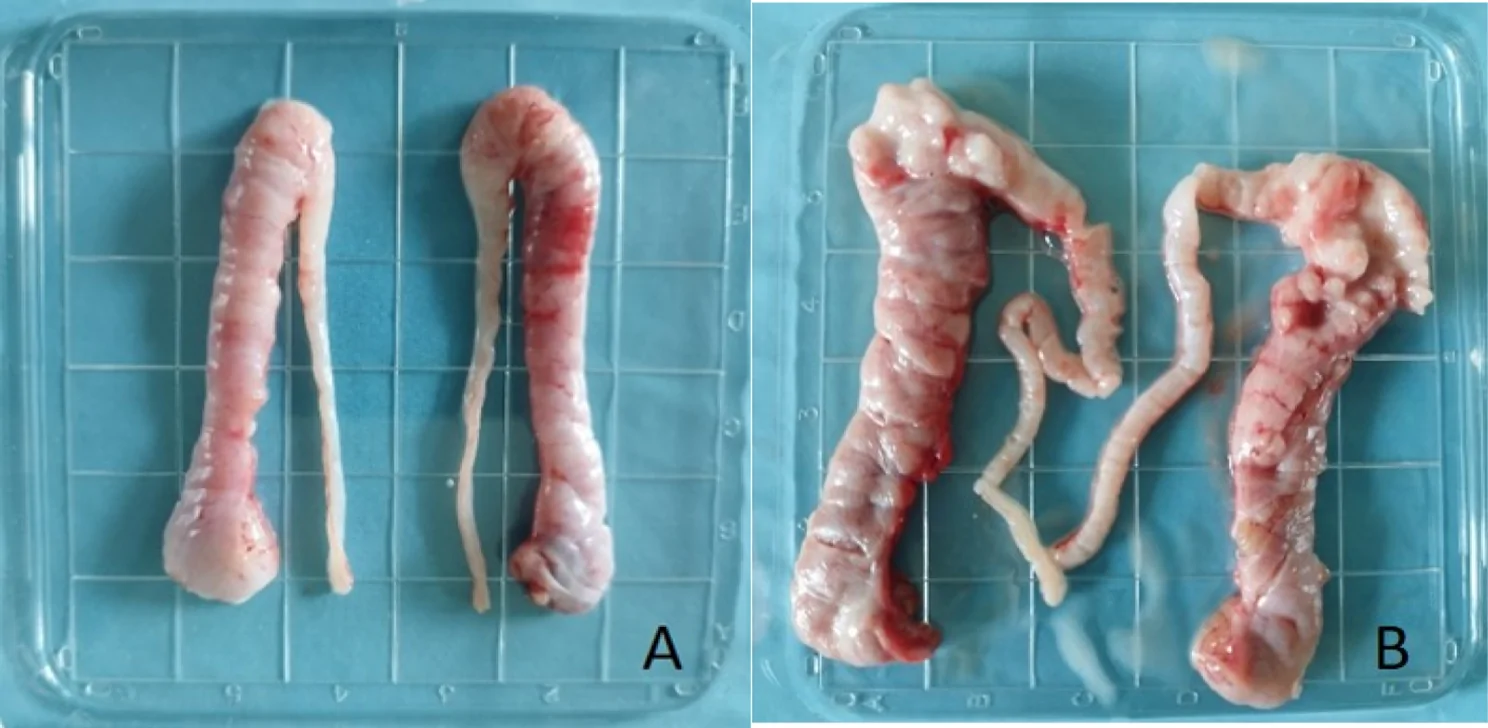
Canine epididymis and ductus deferens before (**A**) and after (**B**) incisions

The cauda epididymis and ductus deferens were separated from each testis and placed in a dry Petri dish. Spermatozoa were recovered using a float-up method at 37 °C [43]. Multiple incisions were made in the epididymis and ductus deferens while avoiding blood vessels, allowing spontaneous release of spermatozoa (Fig. 2).

Seven milliliters of coconut water extender (50% v/v coconut water, 25% v/v distilled water, and 25% v/v 5% anhydrous monosodium citrate solution) were added to the Petri dish, which was then covered with a metal plate to protect spermatozoa from light exposure. Coconut water was obtained from certified organic green coconuts (Alnatura Bio7, GmbH, Philippines). After 45 min, the sperm-containing medium was aspirated into a test tube and subjected to sperm evaluation.

## Sperm evaluation

### Concentration and motility

Sperm concentration and kinematic parameters were assessed using a computer-assisted sperm analysis (CASA) system (Sperm Class Analyzer – SCA, Microptic S.L., Barcelona, Spain) with a Makler counting chamber (10 μL sample volume). A minimum of 30 microscopic fields was evaluated. At least 30 microscopic fields were analyzed for each sample.

The following parameters were recorded: sperm concentration (×10^6^ /mL), total motility (TM, %), progressive motility (PM, %), curvilinear velocity (VCL, µm/s), straight-line velocity (VSL, µm/s), average path velocity (VAP, µm/s), linearity (LIN, %), straightness (STR, %), amplitude of lateral head displacement (ALH, µm), beat cross frequency (BCF, Hz). The CASA analysis temperature was maintained at 37 °C. The following settings for canine semen were used: frame rate − 60 fps, frames acquired − 30–45, chamber depth − 10 µm, sperm concentration for analysis ~30–60 × 10^6^ /mL, fields analyzed ≥ 30, VCL minimum / immotile threshold −20 µm/s, VAP threshold for motile ~25 µm/s, progressive threshold VAP > 50 µm/s + STR > 80%.

### Morphology

Sperm morphology was evaluated using the SCA Morphology module (Microptic, Spain). Samples were stained with SpermBlue for 2 min and at least 50 microscopic fields were analyzed.

### Viability

Sperm viability was assessed using eosin-nigrosine staining. A minimum of 500 spermatozoa were counted under oil immersion at 400× magnification. Unstained spermatozoa were considered viable, whereas pink or red cells were classified as non-viable.

### Semen processing, vitrification, and warming

Following initial evaluation, samples were centrifuged at 581 × g for 10 min at 37 °C. The supernatant was removed and spermatozoa were resuspended in the same coconut water extender supplemented with 20% (v/v) egg yolk (pH 6.88; osmolarity 310 mOsm/kg). Final sperm concentration was adjusted to 100 × 10^6^ spermatozoa/mL (final osmolarity 718 mOsm/kg).

**Fig. 3:**
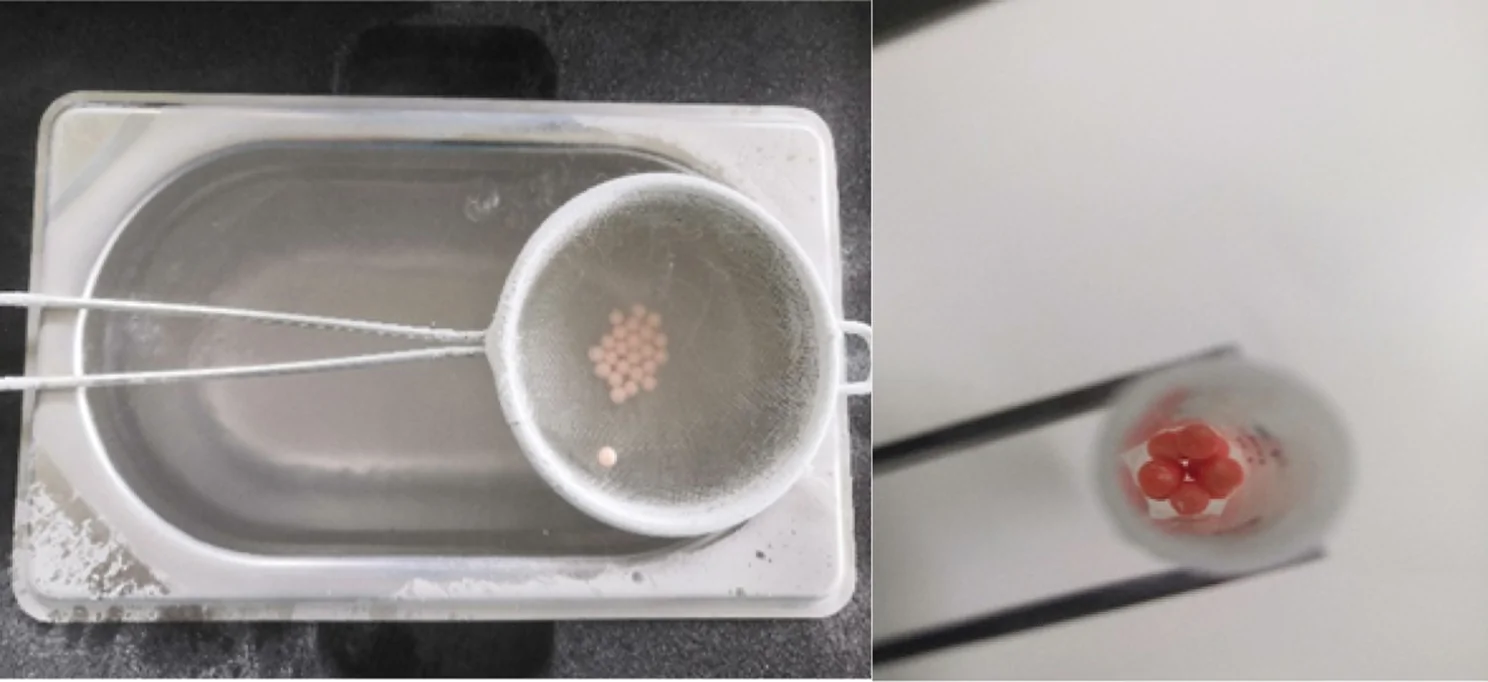
Solidified canine epididymal sperm suspension

Samples were equilibrated at 4 °C for 60 min and vitrified by directly dropping 33 μL aliquots from a height of 10 cm onto a stainless-steel strainer positioned above liquid nitrogen (LN₂) [44]. Solidified pellets were transferred into cryotubes and stored in LN₂ for 7 days (Fig. 3).

Left- during the vitrification process into the strainer, placed into liquid nitrogen; Right- sperm pellets after transfer into cryotube.

Devitrification was performed using two warming regimens:

1. Three pellets placed into 0.5 mL CaniPlus AI (Minitüb, Germany) at 37 °C for 2 min.
2. Three pellets placed into 0.5 mL CaniPlus AI (Minitüb, Germany) at 42 °C for 2 min.

Immediately after warming, spermatozoa were evaluated using the parameters described above.

### Statistical analysis

Statistical analyses were performed using Statistica version 7.0 (StatSoft Inc., Tulsa, OK, USA). Data are presented as mean ± standard deviation (SD). Normality was assessed prior to analysis. Repeated-measures ANOVA followed by Tukey’s post hoc test was used for comparisons. Differences were considered statistically significant at *p* < 0.05.

## Results

The mean volume of the recovered sperm fraction was 0.97 ± 0.23 mL and sperm concentration was 977 ± 178.13 × 10^6^ spermatozoa/mL. Detailed results for viability, motility, and kinematic parameters are presented in Table 1.

**Table 1:** Parameters of fresh and devitrified canine epididymal spermatozoa following warming regimens. Data are expressed as mean ± SD

	Viability, %	Total motility, %	Progressive motility, %	VCL, µm/s	VSL, µm/s	VAP, µm/s	LIN, %	STR, %	ALH, µm	BCF, Hz
Fresh semen	84.71± 17.32 A	69.46± 19.14 A	43.31± 15.39 A	191.21± 26.63 A	128.2± 16.33 A	146.3± 16.20 A	64.30± 5.96 A	89.83± 5.31 A	5.3± 0.91 A	25.7± 3.90 A
V/W37 °C	47.53± 20.1 Ba	40.13± 15.56 Ba	23.41± 8.95 Ba	179.4± 13.7 Aa	123.81± 18.5 Aa	143.2± 13.7 Aa	62.78± 4.95 Aa	85.68± 6.73 Aa	5.59± 1.17 Aa	23.2± 3.6 Aa
V/W42 °C	30.23± 16.18 Bb	24.33± 7.36 Bb	16.71± 8.25 Ba	180.3± 12.4 Aa	121.3± 16.5 Aa	142.9± 11.6 Aa	63.8± 9.3 Aa	84.38± 3.83 Ba	5.49± 0.71 Aa	22.6± 2.8 Aa

V/W 37 °C = vitrification followed by warming at 37 °C; V/W 42 °C = vitrification followed by warming at 42 °C. Different uppercase letters (A, B) within the same column indicate significant differences between fresh and devitrified samples (*p* < 0.05). Different lowercase letters (a, b) within the same column indicate significant differences between the two warming regimens (*p* < 0.05)

Fresh semen exhibited significantly higher (*p* < 0.05) viability, total motility, progressive motility, and normal morphology compared with vitrified samples regardless of warming temperature

Post-warming sperm viability differed significantly between warming regimens. Samples warmed at 37 °C showed higher viability (47.53±20.10%) than those warmed at 42 °C (30.23±16.18%) (*p* < 0.05)

A similar pattern was observed for total motility, with higher values after warming at 37 °C (40.13±15.56%) compared with 42 °C (24.33±7.36%) (*p* < 0.05)

Progressive motility decreased following vitrification in both groups. Although values were numerically higher in the 37 °C group (23.41±8.95%) than in the 42 °C group (16.71±8.25%), the difference was not statistically significant (*p* > 0.05)

No significant differences (*p* > 0.05) were detected between fresh and vitrified samples in most velocity parameters (VCL, VSL, VAP, LIN, ALH, and BCF) for either warming regimen. A significant difference was observed only in STR

### Morphology

The percentage of morphologically normal spermatozoa was significantly higher (*p* < 0.05) in fresh samples compared with vitrified samples. After devitrification, samples warmed at 37 °C demonstrated a higher proportion of normal spermatozoa (72.91±5.72%) than those warmed at 42 °C (68.51±4.92%) (*p* < 0.05).

The most frequently observed morphological abnormalities after vitrification included cytoplasmic droplets and bent or coiled tails (Table 2).

**Table 2:** Morphological abnormalities of fresh and devitrified canine epididymal semen using coconut water extende. Data are expressed as mean ± SD

Morpological Defects	Fresh semen	V/W37 °C	V/W42 °C
Head	1.13 ± 0.89 A	2.12 ± 1.96 Aa	3.35 ± 3.13 Ba
Midpiece defects	1.28 ± 0.68 A	2.68 ± 2.03 Ba	3.51 ± 2.78 Ba
Proximal droplet	3.18 ± 1.01 A	4.13 ± 2.18 Aa	5.11 ± 2.12 Ba
Distal droplet	2.96 ± 1.23 A	5.89 ± 2.88 Ba	6.27 ± 3.14 Ba
Bent tail	2.07 ± 1.75 A	5.93 ± 3.78 Ba	6.04 ± 2.67 Ba
Coiled tail	4.69 ± 2.83 A	6.24 ± 3.11 Aa	7.11 ± 4.88 Aa
Normal spermatozoa	84.68 ± 6.76 A	72.91 ± 5.72 Ba	68.51 ± 4.92 Bb

V-vitrification; W-warming. Uppercase letters (A, B) indicate significant differences in fresh semen samples compared to samples after devitrification (*p* < 0.05). Lowercase letters (a, b): mark sighnificant differences in semen quality after devitrification comparing both warming regimens (*p* < 0.05)

## Discussion

Conventional canine semen cryopreservation has long been used to enhance the reproductive potential of stud dogs [16]. However, this method is frequently associated with substantial sperm mortality and increased morphological abnormalities after thawing [45, 46]. Consequently, considerable research has focused on developing alternative preservation strategies, including both conventional and ultra-rapid freezing techniques for epididymal spermatozoa [20, 43, 47].

Vitrification has been successfully applied to canine spermatozoa during the past decade [1, 16–24], yet optimization of protocols remains necessary. Furthermore, epididymal spermatozoa differ from ejaculated spermatozoa in several biological characteristics, including membrane composition, metabolic activity, and exposure to seminal plasma. These differences may influence their response to vitrification and warming procedures. Consequently, protocols developed for ejaculated semen cannot always be directly extrapolated to epididymal spermatozoa, emphasizing the importance of species- and sample-specific optimization.

In the present study, epididymal spermatozoa were recovered using the float-up method. Although this technique may introduce minor contamination with blood cells or cellular debris, it was selected due to its simplicity and previously demonstrated satisfactory outcomes in terms of viability, motility, and morphology [43, 48]. Sperm retrieval using the coconut water extender yielded quality parameters comparable to or exceeding previously reported values for epididymal spermatozoa [20], indicating that this medium is suitable for recovery and handling of epididymal semen.

The present study specifically evaluated the influence of the warming regimen on vitrified canine epididymal spermatozoa. Coconut water was selected as an extender component because it is a natural, inexpensive, and readily available substance with strong antioxidant capacity. To minimize compositional variability, natural green coconut water without additives from controlled organic production was used. The post-warming semen characteristics observed in this study were comparable to or higher than previously published data [20], supporting the potential suitability of coconut water as a component of vitrification extenders for canine epididymal spermatozoa.

Egg yolk is a commonly used cryoprotective additive that protects spermatozoa against cold shock and stabilizes cell membranes during freezing and thawing [1, 16, 39–41]. In the present study, egg yolk was used as the principal cryoprotective additive, and acceptable post-warming sperm quality was obtained, supporting its potential role in protecting canine epididymal spermatozoa during vitrification.

Sperm motility is one of the primary indicators of fertilizing potential [49], and values of 30–50% motility in frozen semen are generally considered acceptable [50]. Typically, the insemination dose ranges between 100 and 200 × 10^6^ spermatozoa/mL, with intrauterine insemination performed approximately 96 hours after ovulation. Lower sperm concentrations have also been used; however in those cases reduced pregnancy rates were observed [51]. Although vitrification significantly reduced motility compared with fresh semen, samples warmed at 37 °C remained within this acceptable range.

Slow warming was used in this study because it has been reported to reduce damage in vitrified canine spermatozoa compared with rapid warming [18]. Higher recovery rates were obtained at 37 °C than at 42 °C, which contrasts with previous findings obtained using ejaculated semen [24]. These findings are consistent with previous studies demonstrating that warming rate is a critical determinant of cryosurvival [42, 52]. During cryopreservation, spermatozoa pass twice through the temperature range between −10 °C and −60 °C, where the plasma membrane is particularly sensitive to damage [16]. Therefore, the warming rate must be carefully coordinated with the cooling rate to minimize structural injury.

The superior sperm quality observed following warming at 37 °C compared with 42 °C may be related to the greater thermal stress imposed by the higher warming temperature. Although rapid warming is generally important for minimizing ice recrystallization during the warming of vitrified samples, the final warming temperature and duration must also be appropriate for the biological characteristics of the spermatozoa and the vitrification medium. Vitrified spermatozoa may remain susceptible to membrane destabilization and osmotic stress during rehydration, and exposure to 42 °C for 2 min may impose a greater thermal burden than warming at 37 °C for the same duration. This may contribute to the lower viability and motility observed in the 42 °C group. The higher proportion of morphologically normal spermatozoa following warming at 37 °C is also consistent with reduced cellular stress during the warming process. However, the present study did not directly assess membrane lipid phase transitions, oxidative stress, mitochondrial function, or intracellular osmotic changes; therefore, these mechanisms should be regarded as possible explanations rather than directly demonstrated causes.

The difference between the present findings and previous observations obtained using ejaculated semen [24] may be explained by differences in membrane composition between ejaculated and epididymal spermatozoa due to the absence of seminal plasma proteins in epididymal semen. These proteins are known to influence membrane remodeling and stability during maturation [53, 54], potentially affecting resistance to thermal stress during warming. Thus, the distinct biological characteristics of epididymal spermatozoa may contribute to their different response to warming temperature.

Our findings are consistent with previous research in ejaculated canine spermatozoa. The authors compared slow warming at 37 °C for 1 min with rapid warming at 65 °C for 2–5 s and reported better sperm quality following warming at 37 °C in their vitrification system [18]. The present study extends these observations to epididymal spermatozoa and demonstrates that, when a slow-warming approach is used, 37 °C for 2 min may provide better post-warming recovery than 42 °C for the same duration. The difference may reflect the distinct biological characteristics of epididymal spermatozoa and the different extender and vitrification conditions used in the present study.

Kinematic parameters are considered useful indicators of fertilizing potential in dogs [55]. While previous reports described decreased velocity characteristics after cryopreservation of epididymal spermatozoa [20], no significant differences were observed in most kinematic parameters between fresh and vitrified samples in the present study. This finding agrees with earlier work from our group [21–24] and further supports vitrification as a viable alternative to conventional freezing.

A limitation of the present study is the relatively small number of animals included. However, acquisition of epididymal samples from clinically healthy dogs suitable for research purposes is inherently limited, and similar sample sizes have been reported in previous canine sperm cryopreservation studies. Future investigations should evaluate fertility outcomes following artificial insemination and explore additional cryoprotective additives and antioxidant supplements to further improve post-vitrification sperm quality. Nevertheless, the present findings indicate that warming temperature is an important parameter in the vitrification of canine epididymal spermatozoa and should be optimized according to the characteristics of the sperm source and vitrification system.

## Conclusions

Vitrification of canine epididymal spermatozoa using a coconut water-based extender supplemented with egg yolk resulted in reduced sperm quality compared with fresh samples; however, the warming protocol significantly affected post-warming recovery. Warming at 37 °C for 2 min preserved viability, total motility, and normal morphology more effectively than warming at 42 °C. These findings indicate that optimization of warming conditions is a critical component of vitrification protocols and support the use of warming at 37 °C as a practical approach for preservation of canine epididymal genetic material. The proposed protocol may contribute to canine germplasm banking and conservation of valuable genetic resources.

## Data Availability

No datasets were generated or analysed during the current study.
